# Spatial Distribution Characterization of Hormonal Metabolites in Morphologically Diverse Jujube Fruits via MALDI-MS Imaging

**DOI:** 10.3390/foods15172977

**Published:** 2026-08-25

**Authors:** Dongye Lu, Yuping Zhang, Yang Wu, Qinghua Pan, Fuyun Lu

**Affiliations:** 1 Institute of Forestry and Pomology, Beijing Academy of Agriculture and Forestry Sciences, Beijing 100093, China; 2 Key Laboratory of Biology and Genetic Improvement of Horticultural Crops (North China), Ministry of Agriculture and Rural Affairs, Beijing 100093, China; 3 Beijing Engineering Research Center for Deciduous Fruit Trees, Beijing 100093, China; 4 Key Laboratory of Urban Agriculture (North China), Ministry of Agriculture and Rural Affairs, Beijing 100093, China

**Keywords:** *Ziziphus jujuba*, mass spectrometry imaging, spatial distribution, fruit shape, plant hormone

## Abstract

*Ziziphus jujuba* Mill. is an important economic tree species in China with nutritional, ecological and ornamental values, and its fruits show abundant variations in size and shape. Phytohormones regulate fruit morphogenesis, yet the spatial distribution characteristics of endogenous phytohormones in jujube fruits with different shapes remain unclear. In this study, matrix-assisted laser desorption/ionization mass spectrometry imaging (MALDI-MSI) was applied to explore the spatial distribution patterns of phytohormones in round, constricted and long jujube fruits. The results indicated that phytohormones were uniformly distributed in the flesh and pericarp of round and constricted fruits. Certain auxins and gibberellins were undetectable in the outer pericarp of long fruits, while one cytokinin was predominantly accumulated at the fruit base. The differential distribution of phytohormones is likely a potential factor triggering jujube fruit morphological differentiation. This study clarifies the spatial variation in endogenous phytohormones, helping elucidate the regulatory mechanism of jujube fruit shape.

## 1. Introduction

*Ziziphus jujuba* Mill. (Chinese jujube), a species of the genus *Ziziphus* in the family Rhamnaceae, is an economically important fruit tree species native to China, with a cultivation history of more than 3000 years. Owing to its desirable attributes including strong stress resistance, early and high yield, ease of management, rich nutritional value, and wide utilization, Chinese jujube combines both economic and ecological benefits. It has become the most widely cultivated dry fruit tree species in China and serves as a pioneer tree species for rural revitalization [1]. Wild jujube germplasm generally produces round or sub-round fruit, whereas fruit shape has become highly diversified following domestication, resulting in abundant germplasm resources. In agricultural production, the fresh jujube market favors fruit with regular shape, uniform size, and large fruit size, while the processing industry demands consistent fruit morphology to facilitate washing, grading, pitting, and automated production, thereby reducing losses and improving efficiency. Therefore, research on jujube fruit shape is crucial for the genetic improvement and new cultivar breeding of jujube. As hormones are key regulators of fruit shape, investigating their distribution patterns in different fruit shapes will help advance further studies on jujube fruit shape formation.

Plant hormones, also known as endogenous hormones, are ubiquitous trace active substances in plants with vital physiological functions. As key endogenous signaling molecules regulating fruit development, they precisely control cell division, cell expansion, and morphogenesis in fruits through intricate synergistic or antagonistic interactions. The final fruit size is largely determined by cell number and the extent of cell expansion, whereas fruit shape is mainly governed by directional and regional differences in cell division and expansion. Different hormones play distinct roles in these processes. Auxin, gibberellin (GA), cytokinin (CK), brassinosteroid (BR), abscisic acid (ABA), and other phytohormones are all involved in fruit development and coordinately regulate fruit size determination [2].

Auxin regulates fruit setting and fruit size by modulating cell division and cell expansion [3]; for instance, Indole-3-acetic acid (IAA) significantly increases fruit size in grape and cucumber by promoting cell expansion [4,5]. Gibberellins mainly stimulate cell division in fruits and maintain cell elongation [6], and their concentrations are positively correlated with final fruit size and pericarp cells [7]. Exogenous gibberellins promote longitudinal cell elongation, thereby increasing fruit size in woodland strawberry (*Fragaria vesca*) [8]. Cytokinins regulate cell division and cell differentiation [2]. Compared with the small-fruited jujube cultivar ‘Jinsixiaozao’, the large-fruited jujube cultivar ‘Jinkuiwang’ accumulates higher levels of zeatin (ZT) and its nucleoside trans-zeatin riboside (tZR) [9]. High tZT content may promote flesh cell division in large-sized ‘Cuiguan’ pear fruits [10]. Abscisic acid is recognized as a growth-inhibiting hormone that ultimately affects fruit size by suppressing cell division in fruits [11]. Exogenous ABA reduces fruit elongation by inhibiting cell expansion in cucumber [12]. In tomato mutants, decreased ABA content results in reduced fruit size [11], indicating that ABA can also positively regulate fruit size. ABA and salicylic acid (SA) accumulate to higher levels in small-sized apple fruits [13]. Although no direct evidence indicates that jasmonic acid directly affects cell division and changes in fruit size, it has been reported that the transcription factor SlLBD40 (lateral organ boundaries domain 40) acts coordinately with SlMYC2, a key regulator in the jasmonic acid signaling pathway, to regulate fruit expansion [14].

Matrix-Assisted Laser Desorption/Ionization Mass Spectrometry Imaging (MALDI-MSI) is an advanced analytical technique in spatial metabolomics that combines mass spectrometry with imaging software. It enables the visualization of the spatial distribution of molecules in samples without the need for specific staining or labeling. Its advantage lies in the ability to perform three-dimensional analysis involving qualification, quantification, and localization. At present, this method has been widely applied in medical, food, and botanical fields, including pharmaceutical research [15,16], metabolite distribution in fruits [17,18], and studies on the physiological mechanisms of plant growth, development, or stress responses [19]. For example, Yin et al. [19] compared the differences in stress tolerance-related metabolites between wild and cultivated soybeans and concluded that soybeans lose environmental adaptability during domestication, and resistance can be improved via breeding strategies involving hybridization with wild soybean. Zheng et al. [20] used MALDI-MSI to investigate the dynamic changes in plant hormones in the abscission zone during calyx abscission of Korla fragrant pear, a process critical for determining fruit quality.

Despite advances in understanding phytohormonal regulation of fruit development, critical gaps remain. Most previous studies relied on bulk measurements of entire fruits, losing spatial information on hormone distribution across tissues. Moreover, while auxin, gibberellins, and cytokinins are known to regulate fruit size and shape via cell division and expansion, the spatial heterogeneity of these hormones—and how their differential distribution across fruit regions shapes morphology—remains poorly understood. Furthermore, although MALDI-MSI has been applied to a few fruit samples, systematic multi-hormone imaging in fruits with contrasting morphologies is lacking, particularly in jujube, which exhibits exceptional fruit shape diversity.

To address these limitations, MALDI-MSI was employed to analyze the spatial distribution characteristics of hormonal metabolites, including auxin, GA, ABA, JA, and CK, in jujube fruits of different shapes. This study aimed to (1) map the spatial distribution of key phytohormones across jujube fruit (including flesh, pericarp, and apex); (2) compare spatial hormonal profiles between differently shaped cultivars; and (3) identify region-specific hormonal signatures potentially involved in fruit shape determination. The findings deepen the understanding of the regulatory effects of hormones on jujube fruit shape from a spatial perspective, and provide a theoretical basis for targeted jujube breeding and fundamental research on the formation mechanism of fruit shape traits.

## 2. Materials and Methods

### 2.1. Reagents and Materials

**Figure 1:**
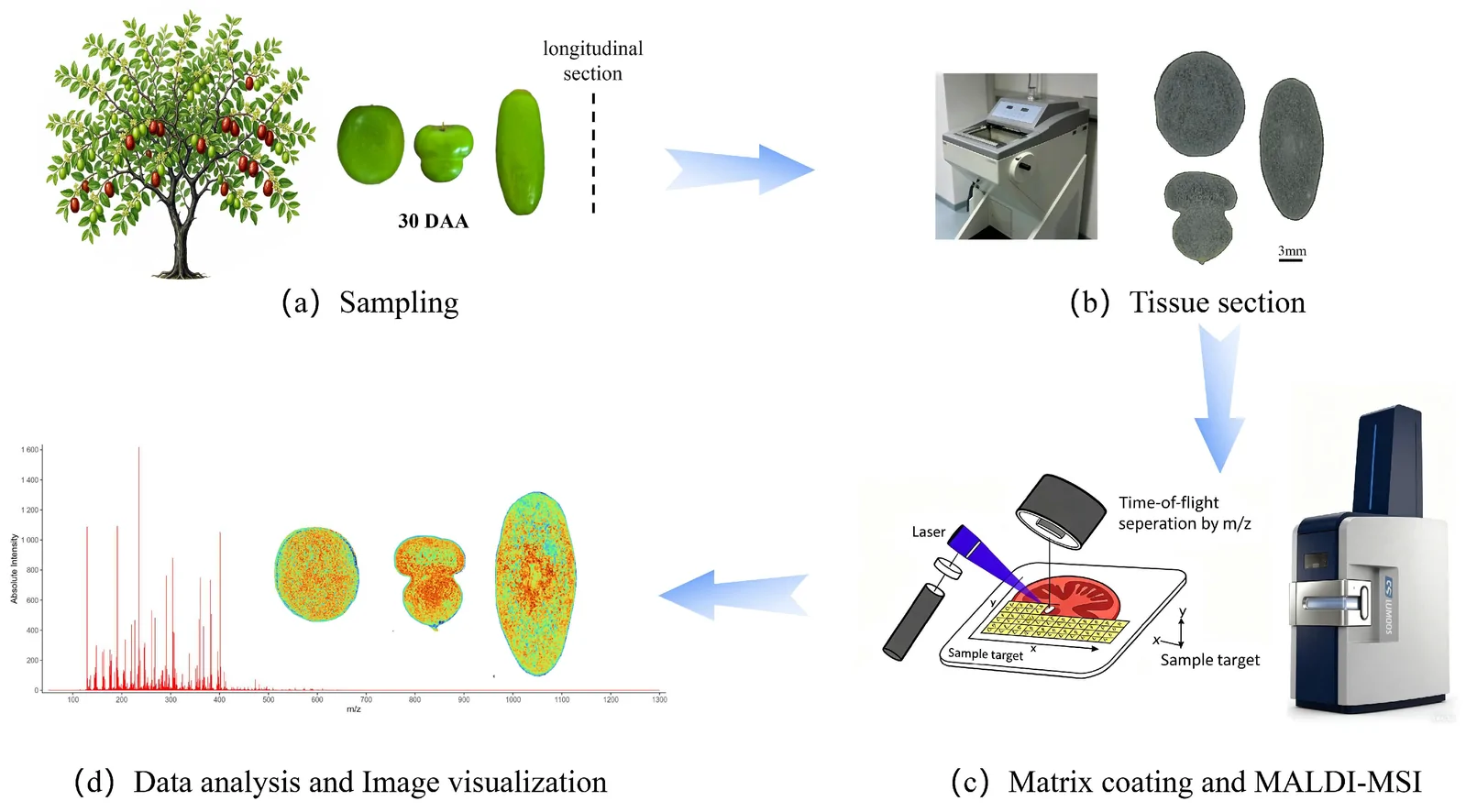
The matrix-assisted laser desorption/ionization MSI (MALDI-MSI) analysis of jujube fruits from different shapes. (**a**–**d**) Experimental procedure of MALDI-MSI for jujube fruits.

The sources and usage of reagents and standards in this study are consistent with those described in our previous research [18]. Three jujube cultivars with distinct fruit shapes—‘Jingyu No. 5’ (round shape), ‘Mopanzao’ (constricted shape), and ‘Mayazao’ (long shape)—were selected as plant materials from the germplasm repository of the Institute of Forestry and Pomology, Beijing Academy of Agricultural and Forestry Sciences. Fruits were collected at 30 days after anthesis (30 DAA), a critical morphogenetic stage when shape divergence is clearly visible across cultivars (Figure 1a). Immediately after collection, the fruits were embedded in 1% sodium carboxymethyl cellulose and frozen, then stored at −80 °C until further analysis. Three biological replicates were performed for spatial metabolomics analysis of each fruit-shape sample.

### 2.2. Sample Preparation and Matrix Spraying

The sample preparation and matrix spraying procedures were as follows: Frozen tissue samples were initially fixed by adding three drops of distilled water during the sectioning step. Subsequently, the tissues were cut into 10 μm thick sections using a Leica CM1950 cryostat (Leica Microsystems GmbHWetzlar, Germany) at −20 °C. Samples from all three different fruit shapes were longitudinally sectioned, with the long pedicel end oriented upward. The prepared sections were mounted on indium tin oxide (ITO)-coated glass slides (Guluo Glass, Luoyang, China) and stored at −80 °C for subsequent analysis (Figure 1b).

Sections from different fruit shapes were grouped and arranged on ITO-coated slides, then placed in a vacuum desiccator for 30 min for drying. The dried tissue sections were subjected to matrix spraying using an HTX TM sprayer (Bruker Daltonics, Bremen, Germany). For hormones such as auxins and abscisic acid, the spraying solution consisted of 1.8 mg/mL N,N-dimethylpiperazine iodide (DMPI) in 80% acetonitrile–20% water. For cytokinins, a 3 mg/mL α-cyano-4-hydroxycinnamic acid (CHCA) solution (70% ACN, 30% H_2_O) was used. The sprayer was set to a temperature of 60 °C, a flow rate of 0.12 mL/min, and a pressure of 10 psi. Each slide received a total of 30 matrix spray cycles, with a drying interval of 6 s between each spray pass.

### 2.3. Mass Spectrometry Imaging

A prototype Bruker timsTOF flex mass spectrometry system (Bruker Daltonics, Bremen, Germany), featuring a 10 kHz smartbeam 3D laser, was employed for MALDI timsTOF MSI experiments. The MALDI MSI parameters were as follows: laser focus, small; laser power, 70%; laser shots, 40; frequency, 10 kHz; laser acquisition mode, single; calibration, external with red phosphorus, <30 ppm. Data acquisition was performed in positive ion mode over a mass range of “*m*/*z*” 50–1300 Da, with a spatial resolution of 50 μm for tissue imaging. Under uniform laser energy, the beam was directed through a grating onto the tissue regions of the target plate. During continuous scanning, laser excitation induced ionization and desorption of analytes from the matrix. The liberated molecules were subsequently analyzed by the mass spectrometer to yield per-pixel information, including “*m*/*z*” values, raw spectral profiles, and peak intensities.

### 2.4. Data Analysis

The raw MALDI mass spectrometry data acquired were imported into SCiLS Lab software (Version 2024a Core, Bruker Daltonics, Bremen, Germany) for smoothing processing and root-mean-square (RMS) normalization to obtain the relative intensity information of different *m*/*z* values at each spatial coordinate, which was then converted into pixels in the imaging heatmaps. The entire imaging regions corresponding to different fruit-shape tissues were selected separately in SCiLS Lab software, and the average mass spectra of each region were obtained. Accurate identification of various hormone metabolites was performed using the MS/MS mode of the timsTOF flex mass spectrometry system. Metabolites in the samples were annotated based on a self-built MWDB (Metabolite Warehouse Database) and public databases including PMhub (https://pmhub.org.cn, accessed on 10 May 2026) and MoNA (https://mona.fiehnlab.ucdavis.edu/, accessed on 10 May 2026). Imaging analysis of all identified characteristic peaks was conducted to obtain the spatial distribution information of hormone metabolites in tissues of jujube samples with different fruit shapes.

## 3. Results and Discussion

### 3.1. Identification of Hormone Metabolites in Samples with Different Fruit Shapes

To clarify the correlation between plant hormone profiles and different fruit shapes, this study employed MALDI-MSI to analyze the spatial distribution of auxins, gibberellins, cytokinins, abscisic acid, and jasmonic acids in young jujube fruits of three distinct fruit shapes at the young fruit stage. Comprehensive metabolite profiles of jujube samples with varying fruit shapes were constructed based on MALDI-MSI data. Supplementary Figure S1 shows the average mass spectra of different fruit shapes.

In this study, raw data of five major classes of hormone-related metabolites were preprocessed and screened. Target MS^2^ peaks were identified by matching with an in-house database combined with the public database PMhub (except for cytokinins, which were analyzed based on average mass spectra), The MS/MS spectra of the representative compounds are provided in the Supplementary Materials (File S1). Subsequently, systematic classification was performed according to hormone types, laying a foundation for subsequent analyses.

A total of 22 metabolites across four hormone classes (excluding isomers) were detected using DMPI as the matrix (Table 1), including 13 auxins (Indole-3-carboxylic acid, *m*/*z* 258.1527; 2-Coumarate, *m*/*z* 261.1523; Indole-3-acetic acid, *m*/*z* 272.1712; 3-Indoleacrylic acid, *m*/*z* 284.1683; 2-oxindole-3-acetic acid, *m*/*z* 288.1632; Indole-3-butyric acid, *m*/*z* 300.1996; Indole-3-lactic acid, *m*/*z* 302.1789; N-(3-Indolylacetyl)-L-valine, *m*/*z* 371.2367; Indole-3-acetyl-L-valine methyl ester, *m*/*z* 385.2524; Indole-3-acetyl-L-aspartic acid, *m*/*z* 387.2037; Indole-3-acetyl glutamic acid, *m*/*z* 401.2109; N-(3-Indolylacetyl)-L-phenylalanine, *m*/*z* 419.2367; Indole-3-acetyl-L-tryptophan, *m*/*z* 458.2476), 5 gibberellins (Gibberellin A9, *m*/*z* 413.2725; Gibberellin A3, *m*/*z* 443.2466; Gibberellin A1, *m*/*z* 445.2623; Gibberellin A19, *m*/*z* 459.2779; Gibberellin A8, *m*/*z* 461.2572), abscisic acid (*m*/*z* 361.2420), and 3 jasmonic acids (Jasmonic acid, *m*/*z* 307.2306; 12-Hydroxyjasmonic acid, *m*/*z* 323.2255; 3-oxo-2-(2-(Z)-pentenyl)cyclopentane-1-butyric acid, *m*/*z* 335.2619).

In contrast, cytokinins were detected using CHCA as the matrix, with 23 metabolites (excluding isomers) identified as proton adducts ([M + H]^+^), including common ones such as Kinetin (*m*/*z* 216.0885), cis-Zeatin (*m*/*z* 220.1198), Dihydrozeatin (*m*/*z* 222.1355), and 6-Benzyladenine (*m*/*z* 226.10927). Detailed information is listed in Table 1. All 45 aforementioned metabolites were acquired in positive ion mode.

### 3.2. Spatial Visualization and Co-Localization of Auxin Metabolites

The regulation of plant fruit shape is a complex and delicate process, in which phytohormones play crucial roles in modulating diverse biological processes. Numerous studies have demonstrated that plant hormones influence fruit morphology by regulating biological processes such as cell proliferation and cell elongation. Auxin is an important hormone affecting fruit shape development; for instance, the IAA content in round peaches is significantly higher than that in flat peaches [21]. Exogenous application of auxin at the anthesis stage of tomato resulted in a marked increase in cell number along the longitudinal axis of the fruit and obvious elongated growth [22].

**Figure 2:**
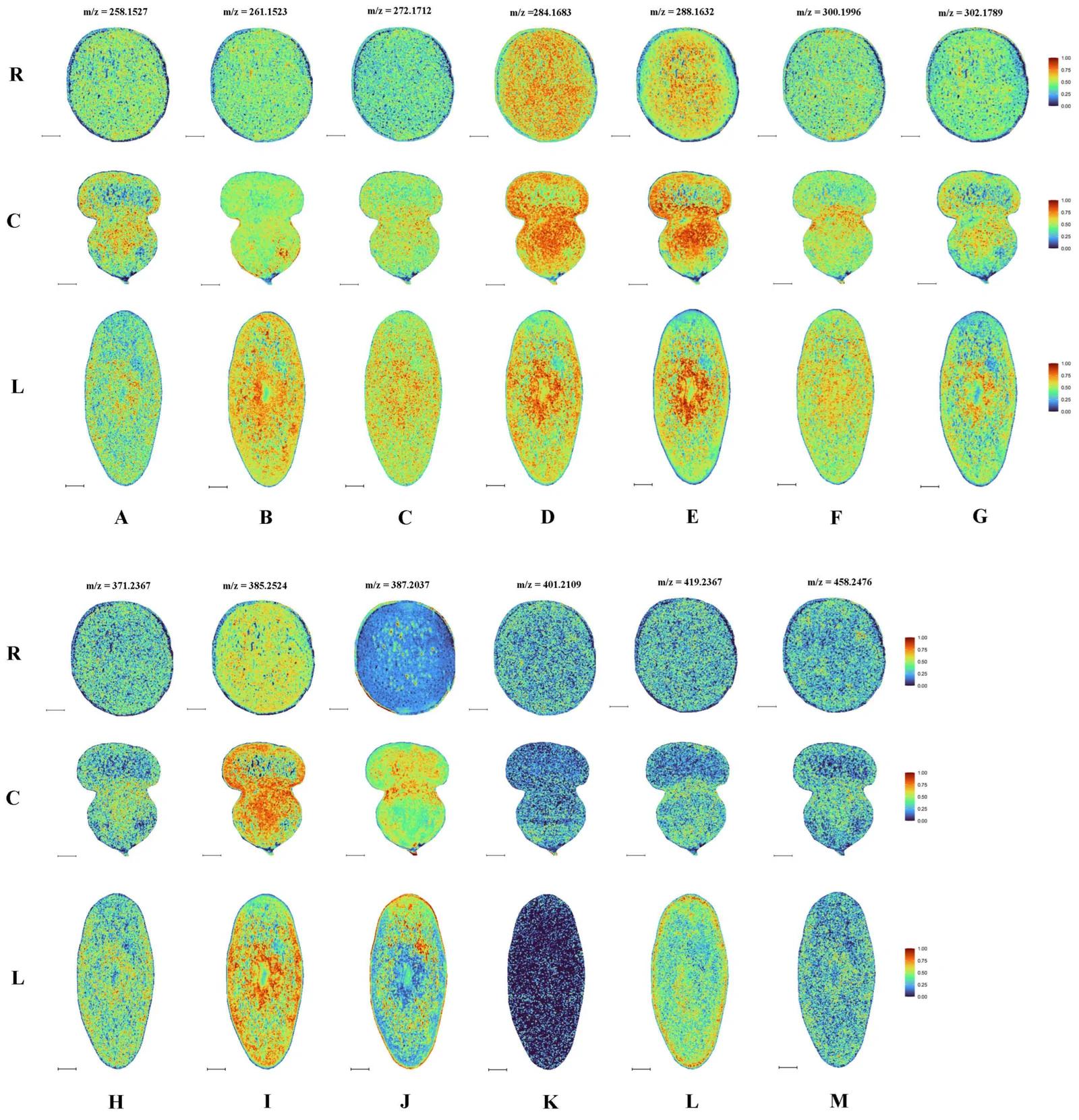
MALDI-MSI reveals preferential accumulation of auxin metabolites in the flesh of jujube fruits of different shapes. (**A**–**M**) refer to 13 auxin metabolites with *m*/*z* values ranging from 258.1527 to 458.2476, respectively. Note: Each row shows composite ion images of different fruit shapes. R: Round; C: Constricted; L: Long. Different colors represent the differences in relative substance intensities within the region. As indicated by the legend on the right, the content of the target substance increases gradually from 0 to 1. The scale bar in the lower left corner represents 2 mm.

Although auxin is known to affect fruit shape, it remains unclear which specific auxin metabolites play decisive roles. Herein, we performed visual analysis of the spatial distribution of auxin metabolites in jujube fruits with different shapes, as shown in Figure 2A–M. Different auxins were distributed almost uniformly in the fruit flesh of different shapes, but their contents varied, showing diverse characteristics reflected by color changes in spatial distribution maps and relative quantitative analysis in the Supplementary Figure S2. The content of IAA in long fruits was higher than that in round and constricted-shaped fruits (Supplementary Figure S2, Table S1), which is consistent with the phytohormone determination results of flat and elongated persimmon fruits reported by Li et al. [23], suggesting a conserved role of IAA in promoting longitudinal growth across species. Among the 13 detected auxin metabolites, 3-Indoleacrylic acid (*m*/*z* 284.1683) (Figure 2D), 2-oxindole-3-acetic acid (*m*/*z* 288.1682) (Figure 2E), and Indole-3-acetyl-L-valine methyl ester (*m*/*z* 385.2524) (Figure 2I) showed the most abundant distributions, indicated by intensified red color in the composite ion map of Figure 2.

More strikingly, certain auxin metabolites display shape-specific distribution characteristics. For instance, N-(3-Indolylacetyl)-L-phenylalanine (*m*/*z* 419.2367) (Figure 2L) displayed an obvious belt-like accumulation along the outer flesh exclusively in long fruits, whereas it was undetectable in the other two shapes, pointing to its potential involvement in shape-specific peripheral tissue expansion. In addition, Indole-3-acetyl-L-aspartic acid (*m*/*z* 387.2037) (Figure 2J) was distributed not only in the fruit flesh, but also in a distinct belt-like pattern along the outer pericarp in all three fruit types, indicating that its function may be more inclined toward fundamental peripheral tissue regulation and this metabolite may be potential candidate to the morphological differences among the three fruit shapes. No obvious accumulation differences in auxin metabolites were observed in the vertical direction, particularly at the fruit apex or constriction sites, among jujube fruits of the three shapes.

In summary, the diversity of auxin metabolite species and their spatial heterogeneity may jointly contribute to the formation of different jujube fruit shapes by influencing local processes such as cell elongation, division, or wall loosening.

### 3.3. Spatial Visualization and Co-Localization of Gibberellin Metabolites

**Figure 3:**
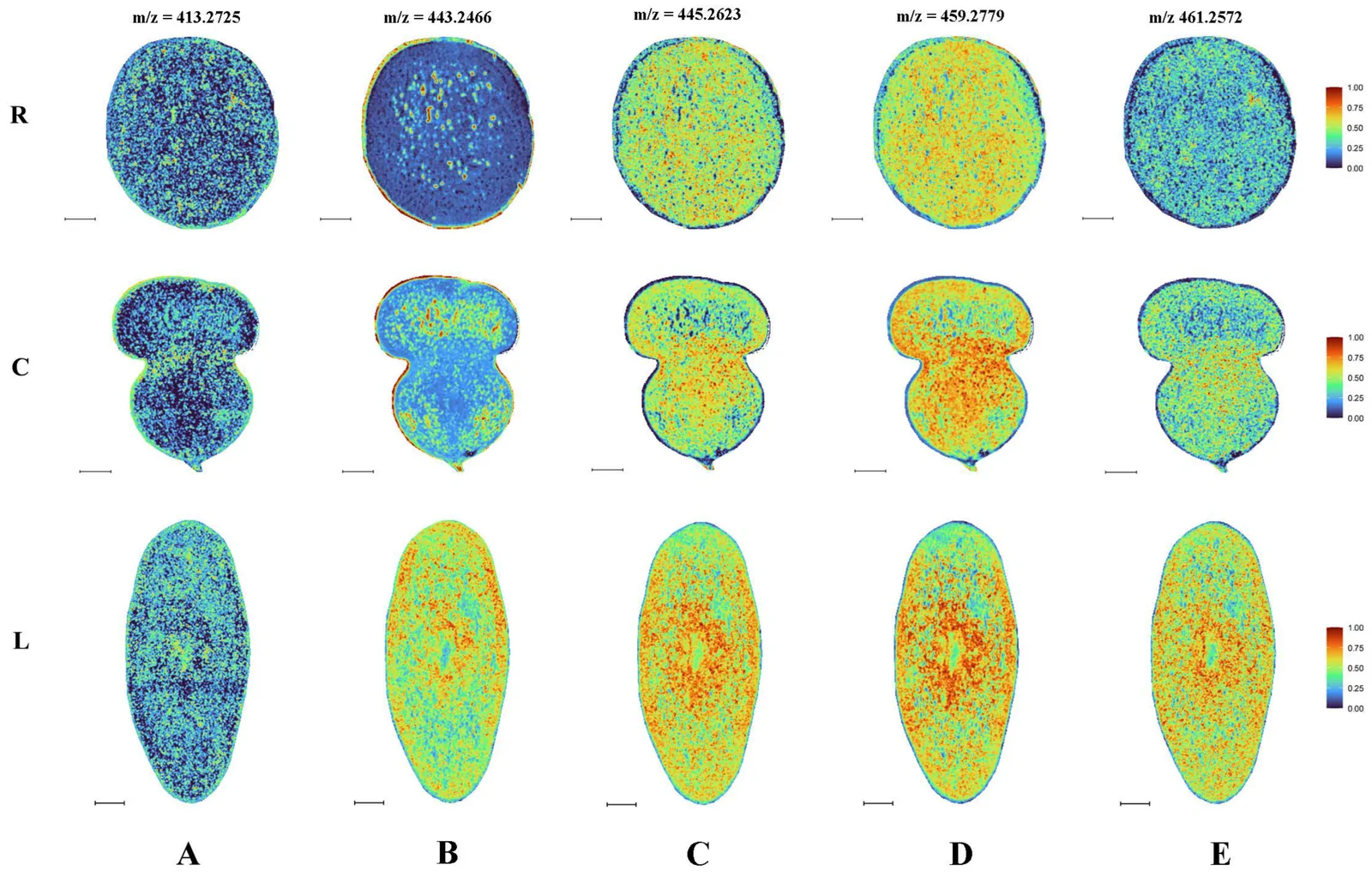
MALDI-MSI reveals preferential accumulation of gibberellin metabolites in the jujube flesh and pericarp with different shapes; (**A**–**E**) refer to 5 gibberellin metabolites with *m*/*z* values ranging from 413.2725 to 461.2572, respectively. Note: Each row shows composite ion images of different fruit shapes. R: Round; C: Constricted; L: Long. Different colors represent the differences in relative substance intensities within the region. As indicated by the legend on the right, the content of the target substance increases gradually from 0 to 1. The scale bar in the lower left corner represents 2 mm.

Gibberellins play a crucial role in fruit morphogenesis, particularly in the regulation of cell proliferation, and exert significant effects on fruit size. It has been reported that treatment with paclobutrazol (PAC), a gibberellin synthesis inhibitor, results in larger and flatter fruits in wild-type tomato, confirming that gibberellins are involved in the regulation of cell expansion during fruit development [24]. In jujube, the overexpression of the gibberellin oxidase gene *ZjCKX5* leads to reduced fruit size [9]. Several detected gibberellins were evenly distributed in the flesh in this study (Figure 3A–E), among which Gibberellin A1 (*m*/*z* 445.2623) (Figure 3C) and Gibberellin A19 (*m*/*z* 459.2779) (Figure 3D) exhibited the highest abundance. The contents of each gibberellin metabolite were higher in long fruits than in round and constricted fruits (Supplementary Figure S2). Gibberellin A3 (*m*/*z* 443.2466) (Figure 3B) was distinctly enriched in the peripheral pericarp of round and constricted jujube fruits, whereas no obvious peripheral accumulation was observed in long fruits, showing an obvious inter-shape difference.

In cucumber, GA_3_ has been shown to restrict longitudinal elongation by inhibiting cell division [5], implying that its localized deposition in the pericarp of round and constricted jujube fruits may similarly suppress radial or peripheral expansion, thereby contributing to a more compact or constricted morphology. Conversely, the absence of GA_3_ accumulation in the peripheral pericarp of long fruits may relieve such suppression, allowing for greater longitudinal growth. This interpretation is further supported by the higher overall gibberellin content in long fruits, which may promote global cell expansion, while the lack of peripheral GA_3_ accumulation enables directional growth along the longitudinal axis. Furthermore, the consistency between our GA_3_ distribution pattern and the findings of Li et al. [23] in persimmon—where GA_3_ levels were higher in flat fruits than in elongated ones—suggests that the role of GA_3_ in modulating fruit shape via peripheral restriction of elongation may be a conserved mechanism across diverse fruit species.

### 3.4. Spatial Visualization and Co-Localization of Abscisic Acid and Jasmonic Acid Metabolites

**Figure 4:**
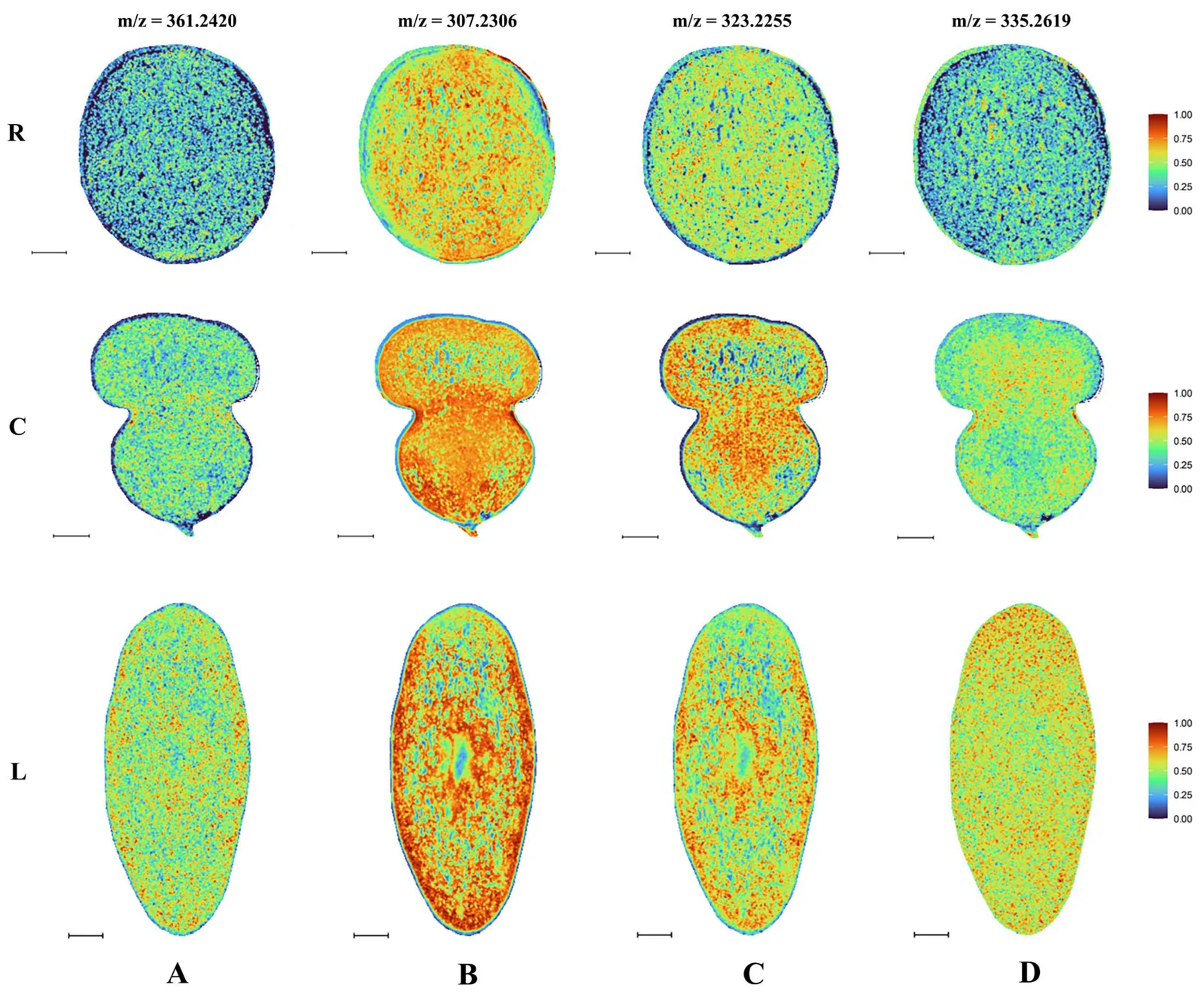
MALDI-MSI analyses of abscisic acid and jasmonic acids preferentially distributed in the jujube flesh with different shapes. (**A**) Abscisic acid at *m*/*z* = 361.2420. (**B**–**D**) Jasmonic acid metabolites at *m*/*z* = 307.2306, *m*/*z* = 323.2255 and *m*/*z* = 335.2619, respectively. Note: Each row shows composite ion images of different fruit shapes. R: Round; C: Constricted; L: Long. Different colors represent the differences in relative substance intensities within the region. As indicated by the legend on the right, the content of the target substance increases gradually from 0 to 1. The scale bar in the lower left corner represents 2 mm.

ABA is recognized as a growth-inhibiting phytohormone, which ultimately affects fruit size by suppressing fruit cell division [11]. Higher accumulation of abscisic acid has been detected in small apple fruits [13]. However, several studies have demonstrated that the length and diameter of cucumber fruits show no significant correlation with endogenous abscisic acid content [5]. In the present study, as shown in Figure 4, abscisic acid (*m*/*z* 361.2420) exhibited a low abundance in fruits of all three shapes (Figure 4A), which appeared to be inconsistent with the above conclusion that small fruits contain a high ABA level (Supplementary Figure S2). This suggests that, under the present experimental conditions, ABA is not a primary determinant of jujube fruit shape or size. Its potential role may be masked by developmental or environmental factors, and further validation is required through time-course or stress-induced experiments.

Jasmonic acid-related metabolites were more abundantly distributed than abscisic acid (Figure 4B–D). Among them, jasmonic acid (*m*/*z* 307.2306) showed the highest accumulation, with obviously higher contents in the pericarp peripheral region than in other fruit tissues (Figure 4B). The results of relative quantification analysis revealed that the contents of jasmonic acid (*m*/*z* 307.2306) and 12-hydroxyjasmonic acid (*m*/*z* 323.2255) in long fruits were significantly higher than those in round and constricted fruits (Supplementary Figure S2).

### 3.5. Spatial Visualization and Co-Localization of Cytokinin Metabolites

**Figure 5:**
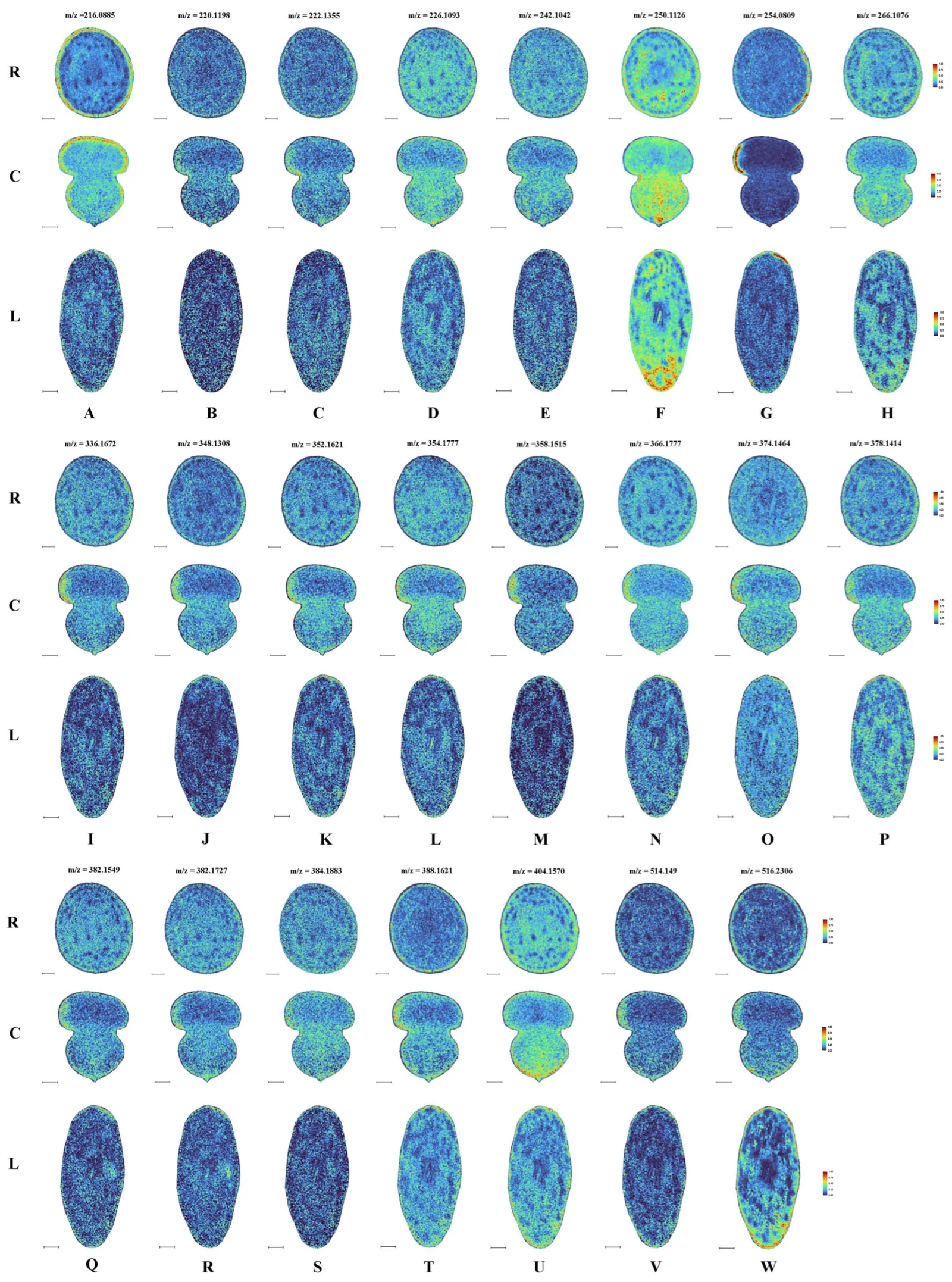
MALDI-MSI analyses of cytokinins preferentially accumulated in the pericarp or apex of jujube fruits with different shapes; (**A**–**W**) refer to 23 cytokinin metabolites with *m*/*z* values ranging from 216.0885 to 516.2306, respectively. Note: Each row shows composite ion images of different fruit shapes. R: Round; C: Constricted; L: Long. Different colors represent the differences in relative substance intensities within the region. As indicated by the legend on the right, the content of the target substance increases gradually from 0 to 1. The scale bar in the lower left corner represents 2 mm.

The shape-dependent distribution patterns of most CK metabolites observed in this study provide further insights into the hormonal regulation of jujube fruit morphogenesis (Figure 5A–M). Kinetin (*m*/*z* 216.0885) exhibited the highest abundance in the pericarp peripheral region across all three jujube fruit shapes, suggesting a fundamental role in cell expansion and morphological maintenance (Figure 5A), which is consistent with its well-established function in promoting cell division and expansion.

More notably, 2-Methylthio-N^6^-isopentenyladenine (*m*/*z* 250.1126) was predominantly enriched in the apical region of long fruits, with relatively low abundance in the upper portion, suggesting a spatial correlation with the apical growth zone (Figure 5F). Whether this metabolite promotes longitudinal elongation by stimulating vertical-oriented cell division at the apex remains a hypothesis that requires functional validation. This observation aligns with previous findings that ZT content is positively correlated with cell number along the longitudinal section of fruits [5], supporting the notion that region-specific CK action can direct differential growth along the longitudinal axis.

Interestingly, while earlier studies have linked higher ZT and tZR levels to larger jujube fruits [9], our results indicate that different CK members may assume distinct and even spatially specialized functions. The apical-specific deposition of 2-Methylthio-N^6^-isopentenyladenine in long fruits, together with the ubiquitous peripheral distribution of kinetin, suggests a functional division among CK metabolites: some provide global growth support throughout the fruit, whereas others exert localized effects that shape the final fruit morphology.

In fact, no single hormone metabolite functions independently during fruit development. In most cases, multiple hormones synergistically regulate fruit morphogenesis. A typical example is the overlapping spatial distribution of auxins and cytokinins, both of which are specifically enriched in tissues adjacent to the pericarp (Figure 2 and Figure 5). Future investigations should focus on the biosynthetic and transport pathways of shape-specifically distributed CK metabolites, particularly 2-methylthio-N^6^-isopentenyladenine, as well as their interplay with other hormones such as auxins and gibberellins in coordinating regional cell division and expansion during fruit development.

### 3.6. Spatial Segmentation Analysis of Target Metabolites

Segmentation analysis provides an overview of imaging mass spectrometry datasets and enables rapid detection of significant features. Statistical methods are adopted to determine the mass spectral similarity of given regions, and pixels with similar mass spectral information are classified into the same category, with each category assigned a specific color [25].

The Spatial Dirichlet Gaussian Mixture Model (Spatial-DGMM) is an image segmentation method independently applied to each mass feature. Spatial-DGMM can accurately segment ion images and distinguish ions with homogeneous and heterogeneous spatial distributions [26]. The parameters were configured as follows: neighborhood smoothing radius (r = 2); maximum number of segmentable regions (k = 10).

**Figure 6:**
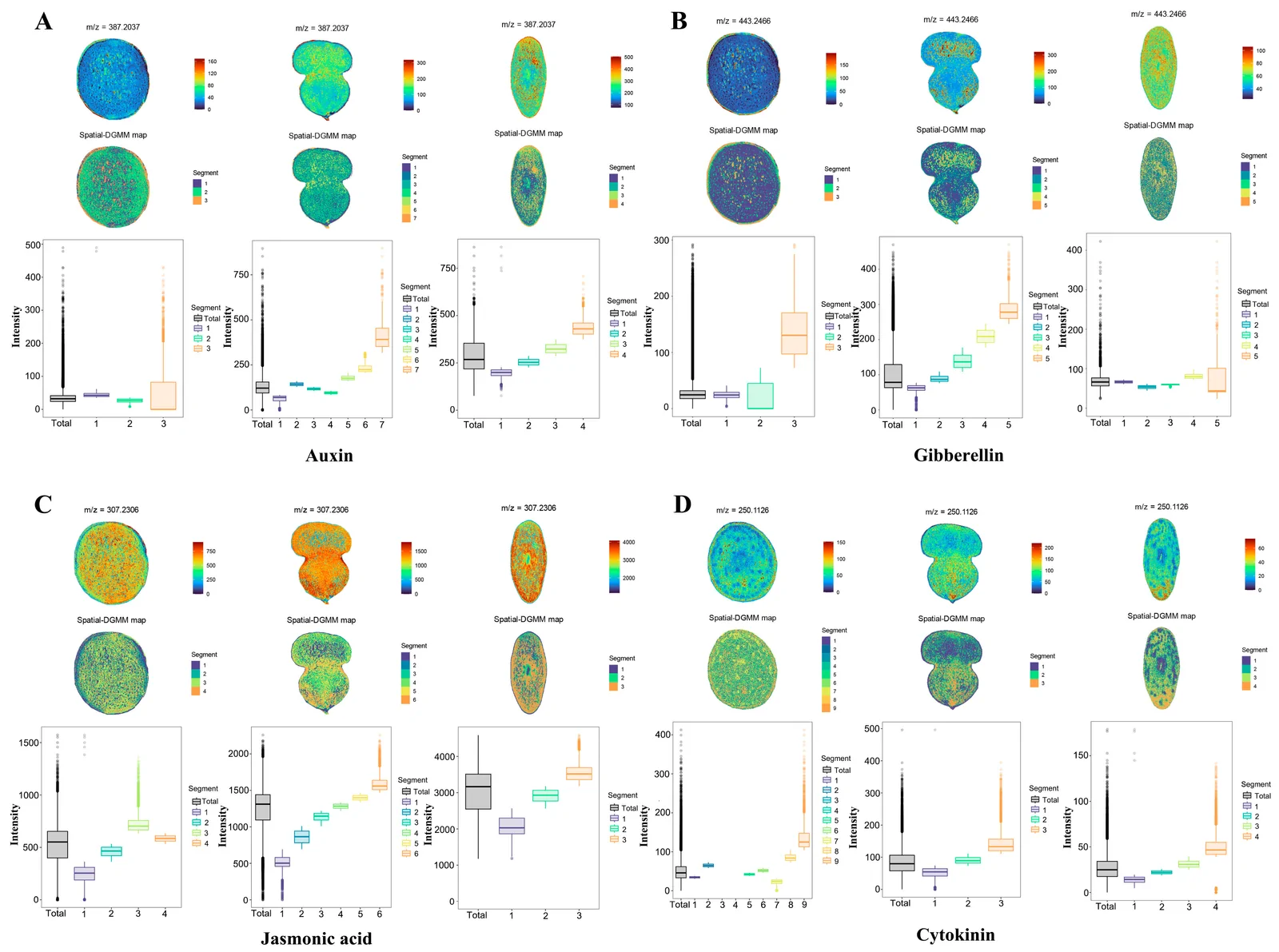
Spatial distribution map of representative hormone metabolites in jujube fruits with different fruit shapes. (**A**) Indole-3-acetyl-L-aspartic acid at *m*/*z* 387.2037. (**B**) Gibberellin A3 at *m*/*z* 443.2466. (**C**) Jasmonic acid at *m*/*z* 307.2306. (**D**) 2-Methylthio-N6-isopentenyladenine at *m*/*z* 250.1126. Note: From top to bottom, each panel sequentially shows the spatial distribution map of precursor ions for spatial DGMM analysis, the spatial DGMM segmentation map (different colors in the map represent distinct spatially segmented regions), and the box plot of ion average intensity (the black box plots summarize the average intensity of the ion across tissues, while the colored box plots summarize the average intensity of the ion in each region identified by spatial DGMM). Different colors represent spatially distinct regions identified by spatial-DGMM, and the same color/number across different samples or ions are not directly comparable.

The Spatial-DGMM segmentation results of representative hormone in this study are shown in Figure 6. Auxins, gibberellins, jasmonic acid, cytokinins and other hormones in the flesh of three different fruit morphologies all exhibited spatial metabolic heterogeneity at the tissue level.The average intensities of Indole-3-acetyl-L-aspartic acid (*m*/*z* 387.2037) and Gibberellin A3 (*m*/*z* 443.2466) were significantly different between the peel and flesh regions (box plot), with higher intensities in the peel than in the flesh (Figure 6A,B). By contrast, jasmonic acid (*m*/*z* 307.2306) showed an opposite trend, with higher ion intensity in the flesh than in the peel (Figure 6C). For the cytokinin 2-Methylthio-N^6^-isopentenyladenine (*m*/*z* 250.1126), enrichment and high ion intensity were observed at the fruit apex of constricted and long fruits (Figure 6D). These results were consistent with the spatial distribution patterns of the above hormone metabolites. It is indicated that the applied imaging mass spectrometry technique can effectively identify metabolic heterogeneity inside tissues, and the proposed method possesses favorable spatial resolution capability.

### 3.7. Co-Localization Analysis of Phytohormone Metabolites

**Figure 7:**
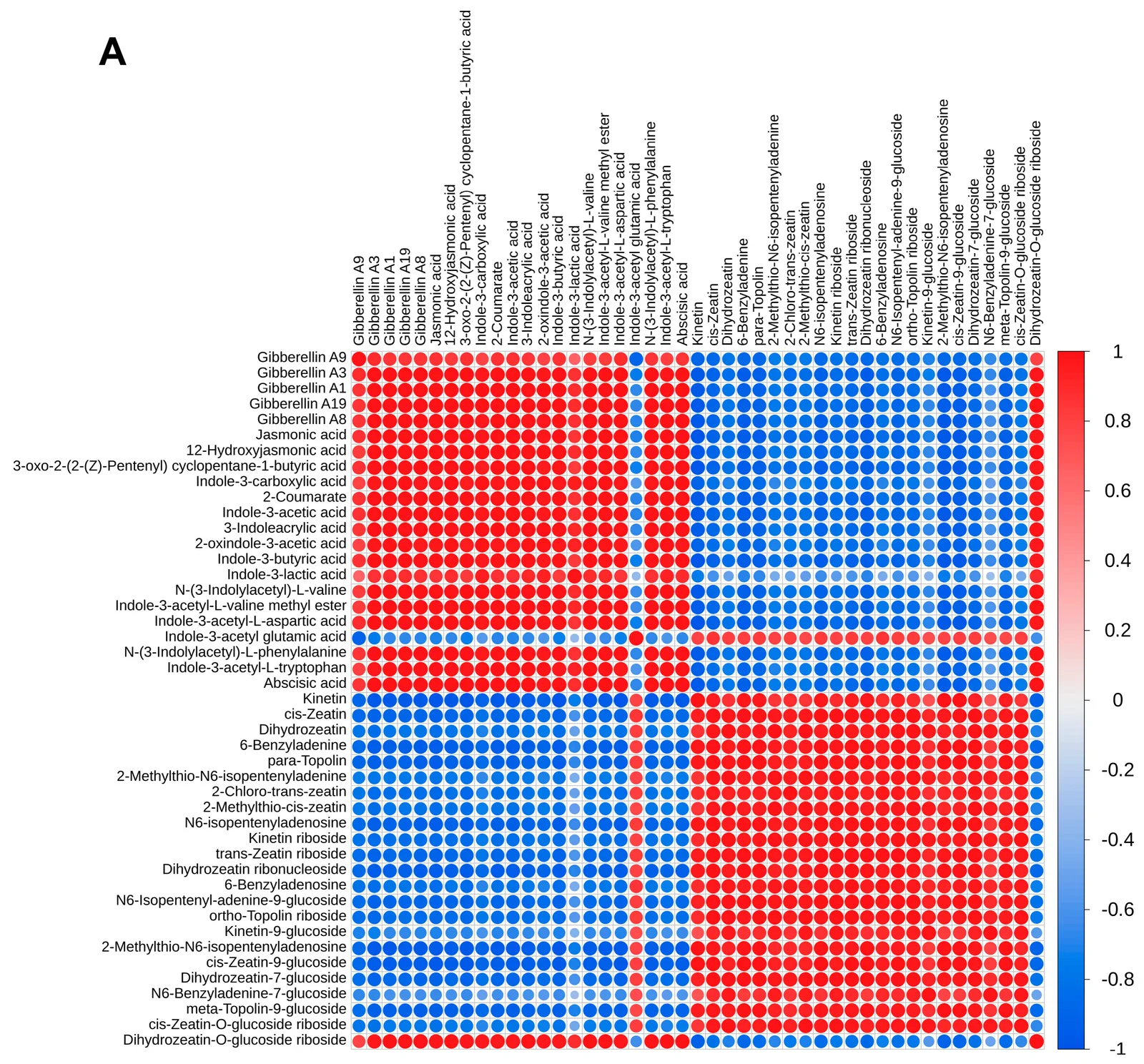

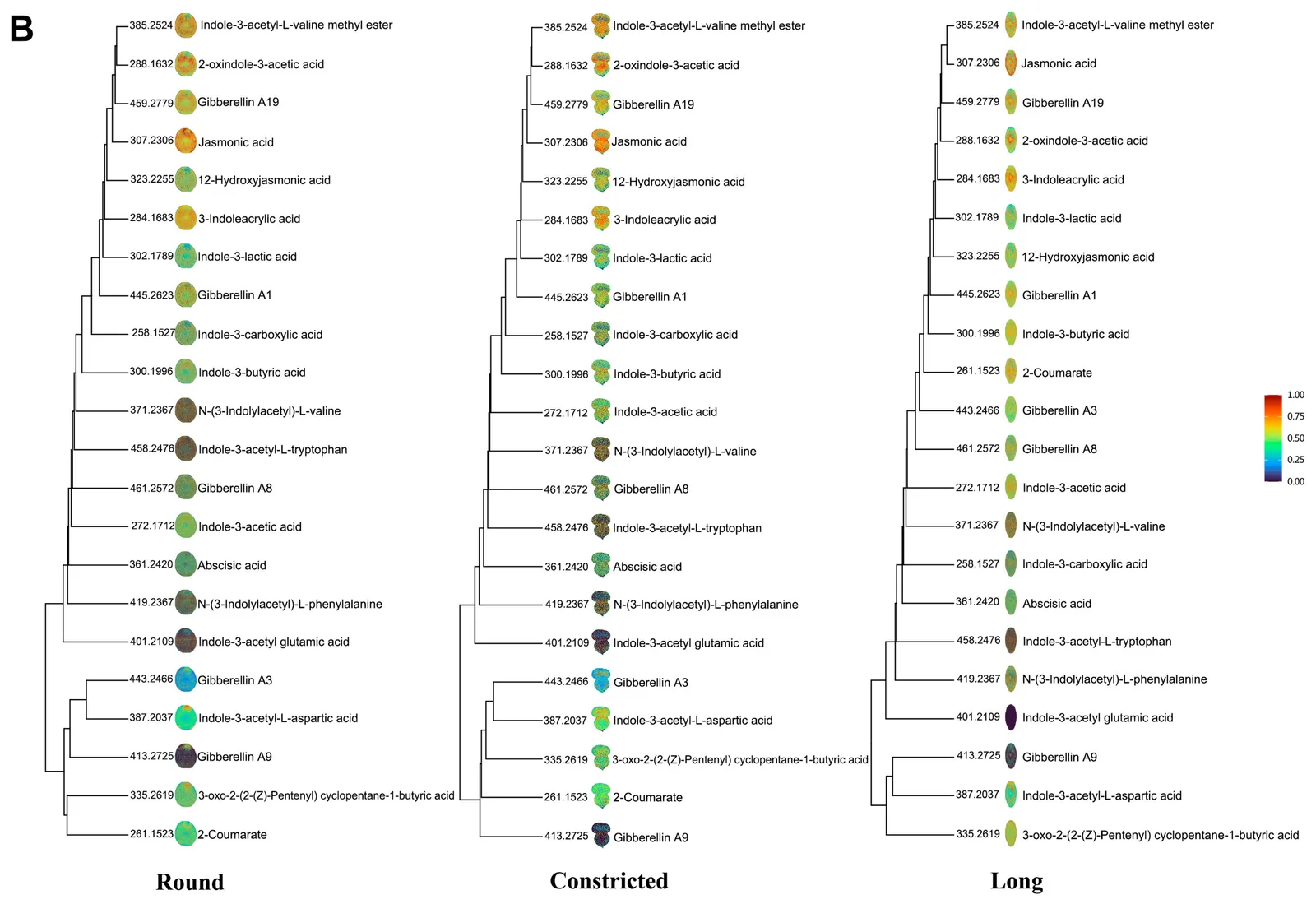
Co-localization analysis of phytohormone metabolites in jujube. (**A**) Correlation matrix heatmap of phytohormone metabolites. In the heatmap, the size of each circle represents the absolute value of the Pearson’s correlation coefficient. Blue indicates a Pearson’s correlation coefficient close to −1, whereas red denotes a coefficient close to 1. (**B**) Co-localization-based phylogenetic trees of phytohormone metabolites (auxins, gibberellins, abscisic acid, jasmonic acid) in jujube fruits with different shapes. Shorter branch lengths on the left indicate higher similarity in spatial distribution among phytohormone metabolites shown at leaf nodes. In the spatial distribution heatmaps of phytohormone metabolites on the right, different colors correspond to varying relative intensities of metabolites in respective regions, with values ranging from 0 to 1 representing a gradual increase in metabolite abundance.

Metabolite co-localization analysis aims to identify metabolites with spatially consistent abundance distribution relative to target metabolites. It explores associations among metabolites and reveals potential molecular interactions via co-localization analysis [27]. To investigate the correlations and spatially specific distributions of phytohormone metabolites in jujube fruits with different shapes, we performed metabolite co-localization analysis using the Pearson’s Correlation Coefficient and Manders’ Colocalization Coefficient based on MALDI-MSI, as reported by Zhao et al. [28] According to the correlation matrix heatmap, auxins (except indole-3-acetyl-L-aspartic acid, *m*/*z* 387.2037), gibberellins, jasmonic acid, and abscisic acid metabolites exhibit strong positive correlations with one another, whereas cytokinins show significant negative correlations with these phytohormone metabolites. Notably, dihydrozeatin-O-glucoside riboside (*m*/*z* 516.2306) is exceptional, displaying positive correlations with the aforementioned phytohormone metabolites (Figure 7A).

The Pearson’s correlation coefficients derived from co-localization analysis were converted into distance matrices, and the Unweighted Pair Group Method with Arithmetic mean (UPGMA) was applied to construct phylogenetic trees for the 45 phytohormone metabolites in each sample [29] (Figure 7B, Supplementary Figure S3). The results revealed that phytohormone metabolites from round-shaped, constricted-shaped and long-shaped jujube fruits shared similar spatial distribution patterns. Three phytohormone metabolites, namely 3-oxo-2-(2-(Z)-pentenyl) cyclopentane-1- butyric acid (*m*/*z* 335.2619), indole-3-acetyl-L-aspartic acid (*m*/*z* 387.2037) and gibberellin A9 (*m*/*z* 413.2725), exhibited nearly identical distributions across the three fruit shapes, predominantly accumulating in the pericarp or near-pericarp regions (Figure 7B). In contrast, gibberellin A3 (*m*/*z* 443.2466) and 2-coumarate (*m*/*z* 261.1523) were uniformly distributed throughout long-shaped fruits, differing from their pericarp-restricted distributions in the other two fruit types, which accounted for their distinct clustering in the phylogenetic trees. Apart from these metabolites, the remaining 40 phytohormones, including the 23 cytokinins shown in the Figure S3, displayed nearly uniform distribution within the flesh tissues in all three fruit shapes.

### 3.8. Study Limitations

It should be noted that the three cultivars examined in this study differ in their genetic backgrounds; consequently, the observed differences in phytohormone spatial distribution cannot be attributed exclusively to fruit shape variation. Genotypic differences among cultivars may affect baseline metabolite levels. Although the present study emphasizes spatial distribution patterns rather than cross-cultivar comparisons of absolute hormone concentrations, this inherent genetic variability constitutes a limitation of our work. Future work, incorporating near-isogenic lines or other genetically uniform populations alongside genetic manipulation approaches, is essential to definitively establish the causal link between fruit morphology and phytohormone distribution. Furthermore, this study was conducted at a single developmental stage (30 DAA). While this time point was deliberately chosen as the critical window for fruit shape differentiation, the spatiotemporal dynamics of phytohormone organization across the entire developmental course remain to be elucidated. Thus, multi-timepoint sampling in future investigations will be essential to fully delineate the developmental trajectories of these spatial patterns and their functional roles in fruit morphogenesis.

## 4. Conclusions

In this study, MALDI-MSI was employed to qualitatively identify and spatially localize phytohormone metabolites (auxins, gibberellins, abscisic acid, jasmonic acid, and cytokinins) in fruit sections of jujube with different fruit shapes. The relative abundances and spatial distribution patterns of these metabolites were compared among jujube fruits of three distinct shapes. The results revealed that auxin (N-(3-indolylacetyl)-L-phenylalanine), gibberellin (A3), and cytokinin (2-methylthio-N^6^-isopentenyladenine) exhibited significant inter-shape differences, suggesting that these metabolites may be spatially associated with the morphological divergence of jujube fruits. MALDI-MSI appears to be a suitable approach for characterizing the spatial distribution of phytohormones in jujube fruits. These findings provide novel insights into the variation rules and spatial distribution traits of endogenous phytohormones in differently shaped jujube fruits, and establishes a spatial framework for fruit shape improvement through the identification of region-specific hormonal signatures linked to particular fruit morphologies.

## Figures and Tables

**Table 1:** The information on metabolites identified by MALDI-MSI in the positive ion mode.

Group	Compounds	Formula	Exact Precursor (Da) *m*/*z*	Molecular Weight (Da)	MS Level	Adduct
Auxin	Indole-3-carboxylic acid	C9H7NO2	258.1527	161.0420	1	M+DMPI
	2-Coumarate	C9H8O3	261.1523	164.0518	1	M+DMPI
	Indole-3-acetic acid	C10H9NO2	272.1712	175.0684	2	M+DMPI
	3-Indoleacrylic acid	C11H9NO2	284.1683	187.0653	1	M+DMPI
	2-oxindole-3-acetic acid	C10H9NO3	288.1632	191.0577	2	M+DMPI
	Indole-3-butyric acid	C12H13NO2	300.1996	203.0892	2	M+DMPI
	Indole-3-lactic acid	C11H11NO3	302.1789	205.0740	1	M+DMPI
	N-(3-Indolylacetyl)-L-valine	C15H18N2O3	371.2367	274.1312	2	M+DMPI
	Indole-3-acetyl-L-valine methyl ester	C16H20N2O3	385.2524	288.1469	2	M+DMPI
	Indole-3-acetyl-L-aspartic acid	C14H14N2O5	387.2037	290.0982	1	M+DMPI
	Indole-3-acetyl glutamic acid	C15H16N2O5	401.2109	304.1054	2	M+DMPI
	N-(3-Indolylacetyl)-L-phenylalanine	C19H18N2O3	419.2367	322.1228	2	M+DMPI
	Indole-3-acetyl-L-tryptophan	C21H19N3O3	458.2476	361.1421	1	M+DMPI
Gibberellin	Gibberellin A9	C19H22O4	413.2725	316.1723	1	M+DMPI
	Gibberellin A3	C19H22O6	443.2466	346.1411	1	M+DMPI
	Gibberellin A1	C19H24O6	445.2623	348.1568	1	M+DMPI
	Gibberellin A19	C20H26O6	459.2779	362.1724	1	M+DMPI
	Gibberellin A8	C19H24O7	461.2572	364.1517	1	M+DMPI
Abscisic acid	Abscisic acid	C15H20O4	361.2420	264.1424	2	M+DMPI
Jasmonic acid	Jasmonic acid	C12H18O3	307.2306	210.1197	1	M+DMPI
	12-Hydroxyjasmonic acid	C12H18O4	323.2255	226.1154	1	M+DMPI
	3-oxo-2-(2-(Z)-Pentenyl) cyclopentane-1-butyric acid	C14H22O3	335.2619	238.1535	1	M+DMPI
Cytokinin	Kinetin	C10H9N5O	216.0885	215.211	1	M+H
	cis-Zeatin	C10H13N5O	220.1198	219.243	1	M+H
	Dihydrozeatin	C10H15N5O	222.1355	221.259	1	M+H
	6-Benzyladenine	C12H11N5	226.1093	225.249	1	M+H
	para-Topolin	C12H11N5O	242.1043	241.259	1	M+H
	2-Methylthio-N6-isopentenyladenine	C11H15N5S	250.1126	249.335	1	M+H
	2-Chloro-trans-zeatin	C10H12ClN5O	254.0809	253.688	1	M+H
	2-Methylthio-cis-zeatin	C11H15N5OS	266.1076	265.262	1	M+H
	N6-isopentenyladenosine	C15H21N5O4	336.1671	335.358	1	M+H
	Kinetin riboside	C15H17N5O5	348.1308	347.326	1	M+H
	trans-Zeatin riboside	C15H21N5O5	352.1621	351.358	1	M+H
	Dihydrozeatin ribonucleoside	C15H23N5O5	354.1777	353.374	1	M+H
	6-Benzyladenosine	C17H19N5O4	358.1515	357.364	1	M+H
	N6-Isopentenyl-adenine-9-glucoside	C16H23N5O5	366.1777	365.384	1	M+H
	ortho-Topolin riboside	C17H19N5O5	374.1464	373.363	1	M+H
	Kinetin-9-glucoside	C16H19N5O6	378.1414	377.352	1	M+H
	2-Methylthio-N6-isopentenyladenosine	C16H23N5O4S	382.1549	381.450	1	M+H
	cis-Zeatin-9-glucoside	C16H23N5O6	382.1727	381.384	1	M+H
	Dihydrozeatin-7-glucoside	C16H25N5O6	384.1883	383.400	1	M+H
	N6-Benzyladenine-9-glucoside	C18H21N5O5	388.1621	387.390	1	M+H
	meta-Topolin-9-glucoside	C18H21N5O6	404.1570	403.389	1	M+H
	cis-Zeatin-O-glucoside riboside	C21H31N5O10	514.2149	513.498	1	M+H
	Dihydrozeatin-O-glucoside riboside	C21H33N5O10	516.2306	515.514	1	M+H

## Data Availability

The original contributions presented in this study are included in the article/Supplementary Materials. Further inquiries can be directed to the corresponding author.

## Supplementary Materials

The following supporting information can be downloaded at: https://www.mdpi.com/article/10.3390/foods15172977/s1, Figure S1: Average mass spectra of imaging regions in jujube tissue samples with different fruit shapes. The x-axis represents the mass-to-charge ratio, and the y-axis represents the average peak intensity in the region after root-mean-square normalization. (A) Hormone metabolites of auxin, gibberellin, abscisic acid and jasmonic acid classes; (B) cytokinin-type substances. Each row corresponds to a different fruit shape. R: Round; C: Constricted; L: Long. Figure S2: Heatmap of relative quantitative contents of identified hormones in jujube samples with different fruit shapes. R1, R2, R3, C1, C2, C3, L1, L2 and L3 represent biological replicates of Round, Constricted and Long-shaped samples respectively. Different lowercase letters (a, b, c) indicate significant differences among groups (Tukey’s HSD, *p* < 0.05). Figure S3: Co-localization-based phylogenetic trees of cytokinin metabolites in jujube fruits with different shapes. Shorter branch lengths on the left indicate higher similarity in spatial distribution among target metabolites shown at leaf nodes. In the spatial distribution heatmaps of phytohormone metabolites on the right, different colors correspond to varying relative intensities of metabolites in respective regions, with values ranging from 0 to 1 representing a gradual increase in metabolite abundance. Table S1: Relative quantification of phytohormone metabolites in jujube fruit samples with distinct morphological types. File S1: MS/MS spectra of representative compounds. Note: In figures, the MS/MS spectrum acquired from the reference standard is shown below the horizontal axis (in red), while that acquired from the sample is shown above the horizontal axis (in black).
