# Supplementary material for: Spatial Distribution Characterization of Hormonal Metabolites in Morphologically Diverse Jujube Fruits via MALDI-MS Imaging
Source: Foods. 2026 Aug 25;15(17):2977. doi: 10.3390/foods15172977 (PMC13565097; doi:10.3390/foods15172977)
Supplement: Supplementary file 1 [file foods-15-02977-s001.zip › File S1.pdf]

Supplementary File S1

MS/MS spectra of representative compounds.

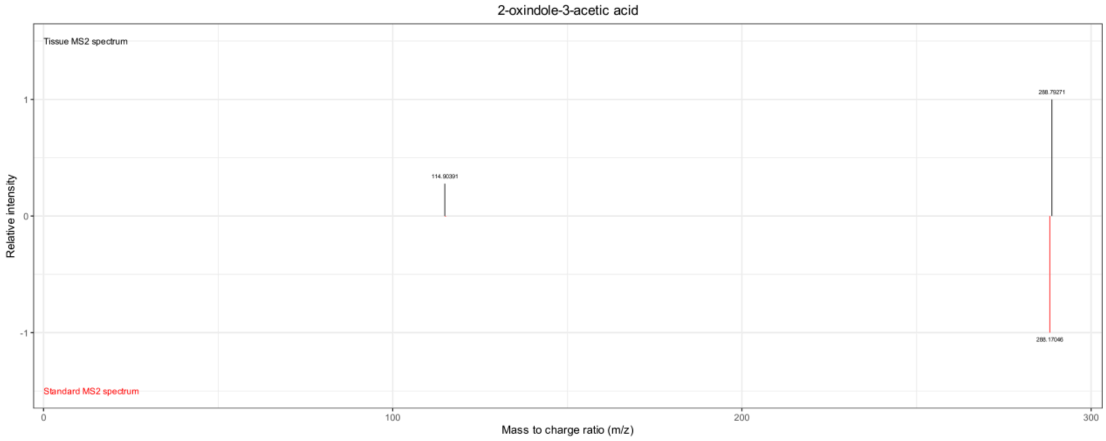

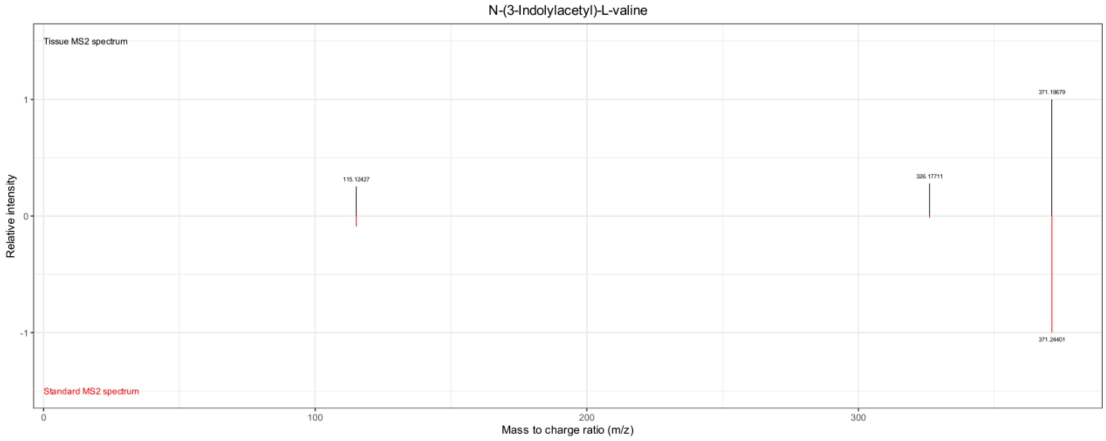

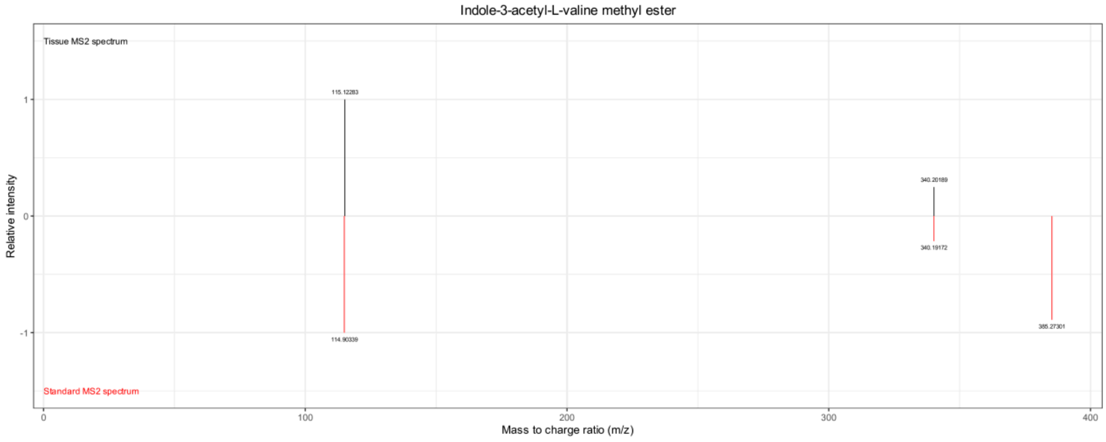

Indole-3-acetic acid

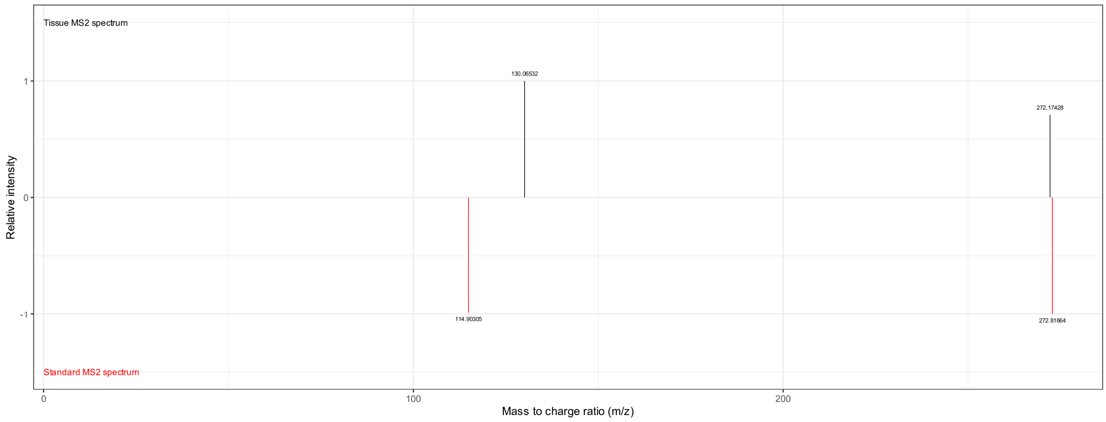

N-(3-Indolylacetyl)-L-phenylalanine

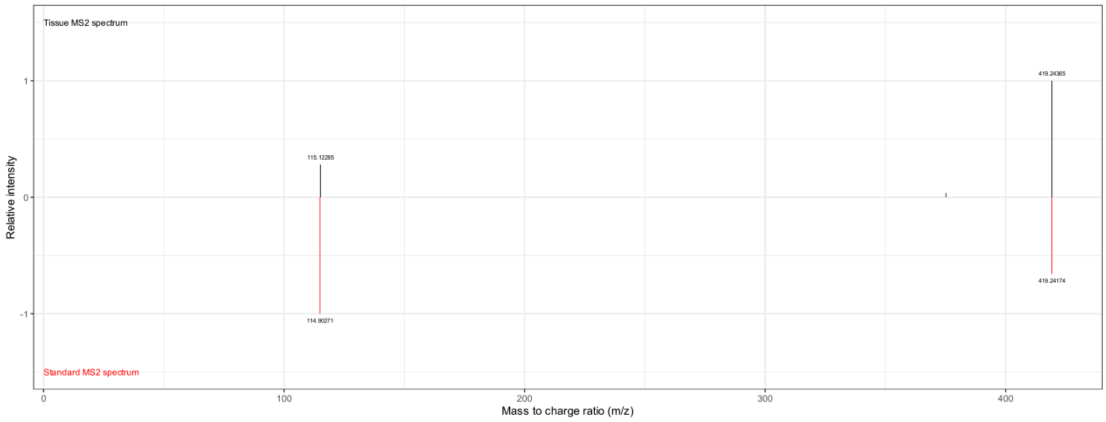

Indole-3-butyric acid

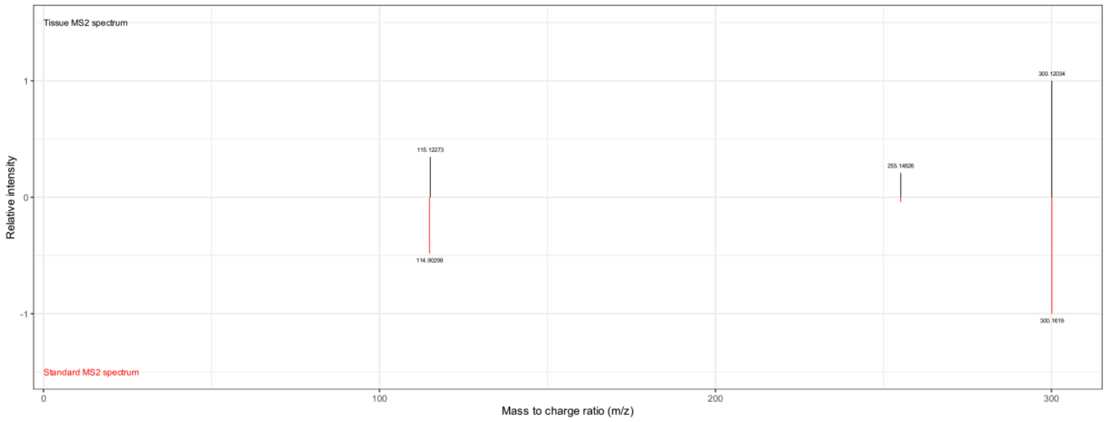

Indole-3-acetyl glutamic acid

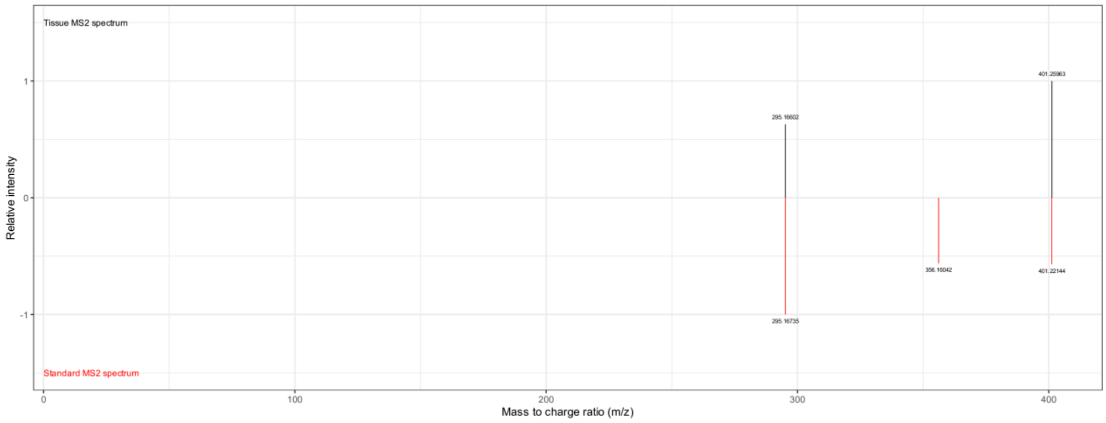

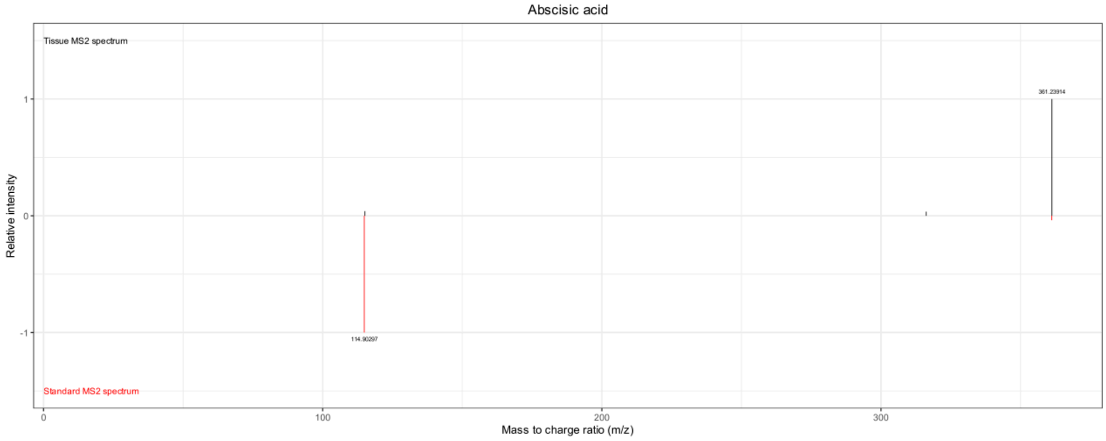

Note: In figures, the MS^2 spectrum acquired from the reference standard is shown below the horizontal axis (in red), while that acquired from the sample is shown above the horizontal axis (in black).
